# Colloidal Quantum Dot Light Emitting Diodes at Telecom Wavelength with 18% Quantum Efficiency and Over 1 MHz Bandwidth

**DOI:** 10.1002/advs.202200637

**Published:** 2022-05-04

**Authors:** Santanu Pradhan, Mariona Dalmases, Nima Taghipour, Biswajit Kundu, Gerasimos Konstantatos

**Affiliations:** ^1^ ICFO‐Institut de Ciències Fotòniques The Barcelona Institute of Science and Technology Castelldefels Barcelona 08860 Spain; ^2^ ICREA—Institució Catalana de Recerca i Estudis Avançats Passeig Lluís Companys 23 Barcelona 08010 Spain; ^3^ Present address: Centre of Nanotechnology Indian Institute of Technology Roorkee Roorkee 247667 India

**Keywords:** colloidal quantum dots, free‐space optical communications, infrared light emitting diodes, LiFi

## Abstract

Developing high performance, low‐cost solid‐state light emitters in the telecom wavelength bandwidth is of paramount importance for infrared light‐based communications. Colloidal quantum dot (CQD) based light emitting diodes (LEDs) have shown tremendous advances in recent times through improvement in synthesis chemistry, surface property, and device structures. Despite the tremendous advancements of CQD based LEDs in the visible range with efficiency reaching theoretical limits, their short‐wave infrared (SWIR) counterparts mainly based on lead chalcogenide CQDs, have shown lower performance (≈8%). Here the authors report on highly efficient SWIR CQD LEDs with a recorded EQE of 11.8% enabled by the use of a binary CQD matrix comprising QD populations of different bandgaps at the emission wavelength of 1550 nm. By further optimizing the optical out‐coupling via the use of a hemispherical lens to reduce optical waveguide loss, the EQE of the LED increased to 18.6%. The CQD LED has an electrical bandwidth of 2 MHz, which motivated them to demonstrate its use in the first SWIR free‐space optical transmission link based entirely on CQD technology (photodetector and light emitter) opening a new window of applications for CQD optoelectronics.

## Graphical abstract

The authors report infrared PbS colloidal quantum dot (CQD) LEDs emitting at 1550 nm with an external quantum efficiency of 18% and 1 MHz bandwidth response via optimizing the composition of the CQD charge supply matrix. They further demonstrate a proof‐of‐concept optical infrared wireless data transfer scheme using a LED and photodetector based entirely on CQD technology.

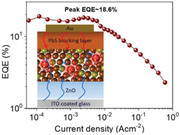

## 1 Introduction

Infrared light‐based communication in the eye‐safe window ≈1500 nm is sought to revolutionize diverse fields of applications—namely, remote control, LiFi, robotics, machine vision, inter‐ and intra‐ machine communications, Internet of things (IoT), medical and biological applications, etc.^[^ ^1^, ^2^, ^3^, ^4^, ^5^ ^]^ To accommodate the requirements for consumer electronics, the underlying optoelectronic components need to offer low‐cost, high‐volume manufacturing, and complementary metal‐oxide semiconductor (CMOS) compatibility. To date, such applications are served by III‐V InGaAs based light emitting diodes (LEDs) and photodetectors that are characterized by high cost, limited production capacity, and lack of monolithic integration to silicon.^[^ ^6^ ^]^ To this end, efforts on developing solution processed optoelectronics for free‐space data transfer and IoT applications have been intensified using organic or perovskite semiconductors, yet those operate in the visible or near‐infrared imposing constraints on optical ambient light interference and eye‐safety regulations.^[^ ^7^, ^8^, ^9^ ^]^ Colloidal quantum dots (CQD)‐based optoelectronics recently emerged as one of the most promising technologies to address the market needs in the eye‐safe short‐wave infrared (SWIR) region, thanks to their low‐cost solution processability, large area manufacturing, band gap tunability, and complementary metal‐oxide semiconductor (CMOS) compatibility.^[^ ^1^, ^10^, ^11^ ^]^

The optical source is of paramount importance for the deployment of such technology and despite compelling performance of CQD‐LEDs in the visible with EQEs over 20%,^[^ ^12^, ^13^, ^14^, ^15^ ^]^ infrared CQD LEDs, particularly based on lead sulfide (PbS) CQDs have remained under performing with a reported EQE of ≈8% at an emission wavelength of ≈1300–1400 nm.^[^ ^16^, ^17^ ^]^ Recently, CQD LEDs based on Ag_2_S (silver sulfide) have been reported to reach an EQE as high as 17% with proper shelling and perovskite matrix incorporation techniques. Although the performance was high at the 1200–1400 nm region, the efficiency drops below 8% for emission wavelength at ≈1500 nm.^[^ ^18^ ^]^ Albeit these were record EQE for CQD LEDs, it remains below the EQE performance of single crystalline III‐V LEDs. Moreover, the use of light at 1550 nm is favorable in terms of eye‐safety yet to date efficient solution‐processed LEDs at this wavelength have remained elusive.

Previously highly efficient SWIR CQD LEDs have relied on the use of perovskite^[^ ^18^, ^19^, ^20^ ^]^ or CQD matrix comprising PbS CQDs with a larger bandgap than of the emitter CQDs.^[^ ^16^, ^17^, ^21^ ^]^ The EQE performance recorded in those prior reports reached ≈8% and was achieved at 1300–1400 nm. Here, we demonstrate highly efficient CQD LEDs emitting at 1550 nm with an EQE of 18%. We report a modified technique that uses two matrix QDs of slightly different bandgaps instead of one matrix QD to form a heterojunction with emitter QDs with a much lower bandgap. The use of a two different bandgap QDs based matrix improved the performance of the device through balanced charge injection, improved light emission, and overall injection efficiency. Utilizing an optimized mixing of 1.75 and 1.35 eV bandgap QD based matrix forming heterojunction with 0.79 eV (excitonic peak ≈1550 nm) bandgap QD based emitters showed an EQE as high as 11.8% while emitting ≈1550 nm. The EQE was further improved to 18.6% utilizing a hemispherical lens attached to the glass substrate of the device to reduce the optical waveguide loss.

## 2 Results and Discussion

**Figure 1:**
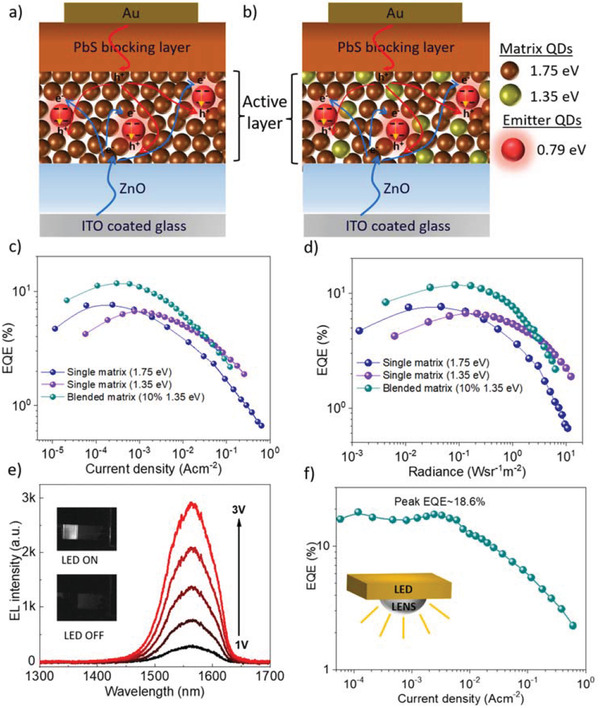
Performance of LED devices: a,b) schematic of binary and blended matrix CQD LED devices. Binary devices comprised of high bandgap matrix and low bandgap emitter QDs. The charges are transferred from matrix to emitter to get the light emission from the emitter sites in the blend (a). Blended matrix‐based devices were formed with matrix QDs of two different bandgaps along with the emitter QDs. The matrix of mixed nature controls the charge injection and charge transfer to the emitter sites and hence improve the device performance (b). c) EQE of the devices as a function of injection current density. Blended matrix‐based devices showed better performance over binary (single matrix QD) devices. d) EQE as a function of radiance. Blended matrix‐based devices showed better performance for most of the range of device radiance. e) Light emission from the LED devices as a function of applied voltage. Inset shows the picture of LED devices with and without applied voltage captured with infrared camera. f) EQE of the lens attached LED devices as a function of applied current. Inset shows the schematic of the hemispherical lens attached to the substrate of the LED device to reduce the substrate induced optical waveguide loss.

**Figures** **1**a,b show the schematic of a single matrix‐based blend and blended matrix‐based devices. The single matrix devices comprise a matrix of QDs with higher bandgap and emitter QDs with lower bandgap. On the other hand, for the blended matrix, the matrix comprised a blend of two size populations of QDs (i.e., possessing different bandgaps) with defined mixing ratios. The emitter QDs bandgap for blended matrix devices are significantly lower than either of the matrix QDs bandgap so that charge transfer can be effectively facilitated from matrix to emitter. Previously, we had reported that binary devices with high bandgap PbS QDs as matrix and lower bandgap PbS as emitter show significantly higher photoluminescence quantum yield (PLQY) and EQE compared to emitter only devices.^[^ ^16^ ^]^ A higher bandgap matrix showed better performance and a lower efficiency droop in the high radiance region due to a balanced charge carrier injection.^[^ ^17^ ^]^ We have extended this idea to the emission of 0.79 eV emitter‐based devices, though the difference in conduction and valence band offset in this case leads to a decrement in device performance. As a consequence, we have utilized blended matrix‐based device to tune the charge injection and hence the device performance. Figure 1c shows the effect of different matrixes on the EQE of the binary and blended matrix‐based devices. 1.75 eV matrix‐based binary devices showed peak EQE of 7.9% with significantly high efficiency droop in the high injection regime. On the other hand, 1.35 eV matrix‐based devices showed a peak EQE of 6.6% with a reduced EQE droop in the high current injection regime (although these devices showed a significantly high efficiency droop in the lower current injection regime). These differences in efficiency droop resulted from the charge imbalance of injected hole and electrons in different regions associated with the difference of conduction and valence band offset between emitter and matrix QDs. Putting emitters in the blended matrix comprised of 1.75 and 1.35 eV QDs significantly improves the EQE as well as the efficiency droop both in lower and higher current injection regimes as shown in Figure 1c. The controlled blended matrix (comprised of 10% 1.35 eV QDs and 90% 1.75 eV QDs) based device showed an EQE as high as 11.8% (10.4±0.7)% (Figure S1, Supporting Information). The effect of different matrices mixing with different ratios is shown in Figures S2 and S3, Supporting Information. A combination of balanced charge injection and improved injection efficiency give rise to the improvement of EQE for blended matrix‐based devices. As a result, the blended matrix‐based devices showed higher EQE compared to 1.35 eV matrix based binary devices in the low radiance regime and with respect to 1.75 eV matrix based binary devices in the higher radiance regime (Figure 1d). The electroluminescence (EL) spectra as a function of applied bias are shown in Figure 1e. Inset shows the infrared emission of these devices as captured with an InGaAs camera (Iberoptics Sistemas Ópticos). In this class of devices, a significant portion of generated light in the active layer is waveguided through the substrate to the edge of the structure leading to increased optical losses. The optical loss due to waveguide through substrates and other layers is estimated at ≈60% as shown in Figure S4, Supporting Information. Thus, LED light outcoupling can be drastically improved by reducing waveguiding loss. Previously, improvement of device EQE by reducing waveguide loss has been demonstrated for various types of visible LEDs.^[^ ^22^, ^23^, ^24^ ^]^ Here, we have addressed this problem by attaching a hemispherical lens with a refractive index matching that of the substrate as shown in Figure 1f (Figure S5a, Supporting Information). By doing so we note a significant improvement of EQE to ≈18.6% setting a new record for SWIR CQD LEDs. The implementation of a lens does not alter the spectral emission of the LED (Figure S5b, Supporting Information).

**Figure 2:**
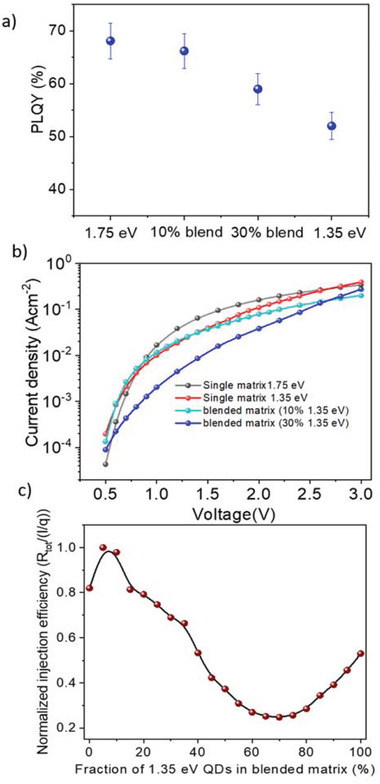
Variation of optical properties and charge injection as a function of blend ratio: a) the PLQY of the binary devices with single matrix and blended matrix. The 1.75 eV matrix‐based devices showed PLQY as high as 68% whereas the 1.35 eV matrix‐based devices showed a PLQY of 52%. PLQY of the blended matrix‐based devices varies systematically in‐between with QD mixing ratio. b) The variation of injection current density as a function of applied voltage with single and blended matrix‐based devices. Blended matrix‐based devices showed lower current injection indicating the control over charge injection through the variation of blend ratio. c) Injection efficiency as simulated with SCAPS, varies as a function of 1.35 eV QD loading in the matrix. Injection efficiency showed a similar trend as observed with device performance.

To understand the underlying mechanism of such an elevated performance, we have performed photoluminescence (PL) characterizations of the binary and different blended matrix‐based films. Figure S6a, Supporting Information, shows the PL spectra of the binary and blended matrix‐based films. Both binary as well as blended matrix devices showed a strong emission corresponding to the emitter QD bandgap. No matrix emission observed for either of these devices suggests efficient charge transfer from matrix to emitters. The PLQY of different devices is summarized in **Figure** **2**a. 1.75 eV matrix based binary devices showed a PLQY as high as 68% compared to 52% for 1.35 eV matrix based binary devices. We posit that these differences arise from the difference of conduction and valence band offsets between emitter and matrix for a 1.75 and 1.35 eV‐based matrix. A relatively deeper well formed by the adjacent matrix and emitter QDs in 1.75 eV matrix based binary mix gives rise to better confinement of charge carriers and subsequently higher radiative recombination. The blended matrix‐based devices showed systematic PLQY in between the values obtained with single matrix QD based blends. A 10% matrix mix (10% 1.35 eV QDs in the blend) and 30% matrix mix (30% 1.35eV QDs in the blend) showed PLQY ≈66% and ≈59%, respectively. The most significant observation from this result is that the PLQY can be tuned by controlling the ratio of matrices in the mix. The PL decay curves as shown in Figure S6b, Supporting Information, also support this with a similar trend. PL decay curves are fitted with double exponential decay times. The faster component corresponds to the emitter recombination whereas the slower one indicates the matrix to emitter charge transfer.^[^ ^16^ ^]^ All the curves showed a similar fast decay component but significantly different slow decay component (the time decay parameters are summarized in Table S1, Supporting Information). Figure 2b shows the injection current density plot as a function of applied voltage with the variation of matrix composition. The blended matrix‐based devices showed a lower injection current compared to the single matrix based binary devices. The injection current in the blended matrix‐based devices can be tuned as a function of blend ratio. The energy band scenarios of the active material ensembles for both single matrix or blended matrix‐based devices are depicted in Figure S7, Supporting Information. Within the blended matrix, the charge injection procedure is complicated compared to the single matrix‐based device as there are a higher number of injection paths available compared to the single matrix‐based devices. Once the charges reach matrix QDs, they will be transferred to the emitter sites irrespective of their band position as described in Section S8, Supporting Information. Moreover, we have computed the injection efficiency of the blended matrix‐based device with SCAPS (Section S9, Supporting Information). The injection efficiency determines the fraction of charge carriers that are injected in the active material, participating in the recombination procedure. The simulation shows the variation of injection efficiency as a function of 1.35 eV QD loading in the blended matrix. The efficiency improves at a very low loading (0–10%) and decreases significantly with higher loading as shown in Figure 2c. Although the simulation conditions used in this case were much simpler than the actual problem, it showed a trend similar to the device performance variation as a function of matrix QD variation. The blended matrix can tune the PLQY as well as control the charge injection efficiency. EQE of an LED depends on the PLQY, injection efficiency, charge balance, etc.^[^ ^17^, ^19^ ^]^ The tuning of these parameters through blended matrix QDs leads to a higher EQE compared to their single matrix‐based counterparts. The strong variation of PLQY and injection efficiency as a function of matrix QD loading ratio suggests their strong influence in determining the device performance.

**Figure 3:**
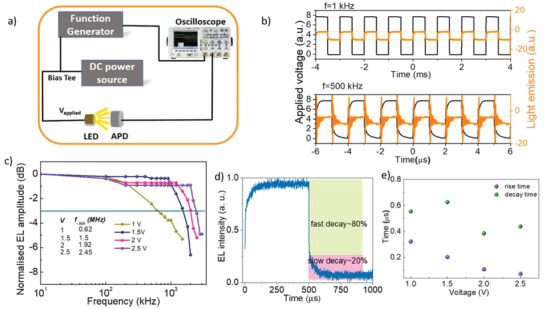
Modulation properties of CQD LED devices: a) schematic of the frequency modulation characterization setup for CQD LED devices. b) Applied voltage bias (black) and related light emission from the LED device (orange) for an applied frequency of 1 and 500 kHz as captured with the oscilloscope. c) Normalized EL amplitude of the LED devices as a function of applied voltage bias. Inset shows the corresponding modulation bandwidth. Modulation bandwidth improves with higher applied bias as a consequence of improved time of flights of the charge carriers. d) EL decay curve of the LED devices comprises of fast decay component (more than 80% weightage) and a slow decay component. Such a faster decay component contributes to the high modulation bandwidth. e) The variation of the faster component of rise and decay time of the EL signal as a function of applied bias. The data were taken for an input signal frequency of 500 kHz. The variation of EL signals as a function of applied bias is shown in Figure S11, Supporting Information. The faster time constants were observed with increased voltage bias due to increased charge carrier time of flights.

To further assess the potential of this class of LEDs for free‐space optical transmission applications, besides efficiency modulation bandwidth is another critical figure of merit. **Figure** **3**a shows the schematic of the experimental setup we used to determine the frequency response and modulation bandwidth of the LEDs (details of the setup is described in the Experimental Section). The effect of applied bias frequency on the electroluminescence signal is depicted in Figure 3b. It shows that the amplitude of the EL signal remains similar for 1 and 500 kHz applied frequencies. To find the modulation frequency, we have scanned the EL amplitude of the LEDs as a function of applied frequency. The modulation bandwidth [corresponding to the 3 dB frequency (frequency at which the amplitude reduces to 70%)] as a function of applied bias voltage is shown in Figure 3c. The modulation bandwidth increases from 0.62 MHz for 1 V bias to 2.45 MHz for 2.5 V bias due to a faster time of flights of the charge carriers. The variation of EL signal as a function of applied bias is shown in Figure S11, Supporting Information. To our understanding, this is the first time such a high modulation width is reported for CQD based infrared LEDs considering slower PL decay time of these materials. To understand the origin of such an improved modulation bandwidth, we have studied the EL decay time. Like the PL, EL also showed two component decays as shown in Figure 3d. The fast component is corresponding to emitter radiative emission and slower component is related to charge transport in the active materials. A closer look at the EL decay curve showed that the faster component comprises more than 80% of the decay curve (Figure 3d). Thus, the faster decay (in sub‐microseconds) of these materials contributed to the improved modulation bandwidth. Figure 3e shows the variation of the faster component of the rise and decay time of EL signal as a function of applied voltage bias. Faster rise and decay time correspond to the increased charge time‐of‐flight in the active materials. The improvement of rise and decay time contributes to the improvement of the modulation bandwidth.^[^ ^25^ ^]^

**Figure 4:**
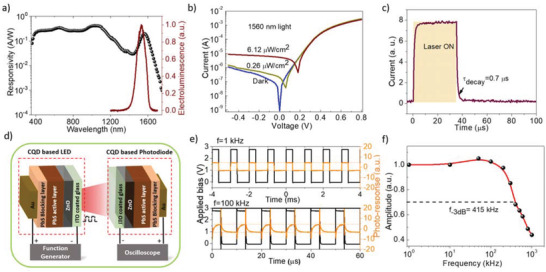
All PbS CQD based integrated device performance for short‐range communications: a) the responsivity of the photodiode based on 1560 nm excitonic peak PbS CQD. The responsivity shows reasonable response throughout the wavelength region of 400 to 1650 nm. The responsivity peak corresponding to the QD excitonic peak matches well with the electroluminescence peak of the LED device. b) Dark and light current–voltage characteristics of the photodiode. Change of photocurrent in the reverse bias observed with the 1560 nm incident light was observed. c) Fast photocurrent decay observed in 1560 nm PbS QD based photodiodes. d) Schematic of all PbS CQD based integrated device designed for short‐range communications. The blended CQD matrix‐based LED was used as the signal transmitter and PbS QD based photodiode was used as the signal receiver. The distance between the transmitter and receiver was 5 cm. e) Frequency response of the receiver photodiode with frequencies 1 and 100 kHz, respectively. The applied voltage bias signal to the LED (black) and signal received with the photodiode (orange) were captured with the oscilloscope. f) Amplitude of the received signal as a function of applied frequency. The modulation bandwidth observed as 415 kHz.

To create an optical link, besides the light source, a photodetector is needed on the receiver side. Recently, PbS QDs with an excitonic peak in SWIR region‐based photodiodes have shown high quantum efficiency.^[^ ^26^ ^]^ Here, we have utilized PbS QDs with an excitonic peak ≈1550 nm (similar to the emission wavelength of the LED devices) to form depleted heterojunction photodiodes following the success of similar structure in PbS QD based solar cells. **Figure** **4**a shows the responsivity of the photodiode over visible to SWIR region. A clear excitonic peak ≈1560 nm with a reasonable responsivity of 0.21 A W^−1^ was observed. The EL emission from the LED devices showed a similar peak position making it an ideal combination for an integrated signal transmitter and receiver‐based communication devices. Further, the current–voltage (*I*–*V*) response curve showed the photo‐response of the devices with incident light intensity (*λ* = 1560 nm) as low as 0.26 µW cm^−2^ confirming the effectiveness of the photodiodes in detecting low optical signal as shown in Figure 4b. The rise and decay time of the photodiode is of utmost importance to determine its efficiency as the signal receiver. The transient photo‐current behavior of the photodiode with an incident light of wavelength 1560 nm is shown in Figure 4c. It shows the decay time of the photodiode is ≈0.7 µs limited by the mobility and the trap density in the active materials. These response times are in the same order of the LED EL and PL decay times making it an ideal component for being a signal receiver for the integrated devices. Figure 4d shows the schematic of a CQD thin film based integrated short‐ranged signal communication system comprising the blended matrix‐based LED devices as signal transmitter and PbS QD based photodiode as signal receiver. The distance between transmitter and receiver was fixed at 5 cm. The response signals as received by the photodiodes with input signals of frequency 1 and 100 kHz, respectively, are shown in Figure 4e. The amplitude of the received is preserved for 100 kHz applied frequency. Figure 4d shows the change of amplitude of the received signal as a function of applied frequency. The 3 dB frequency (modulation bandwidth) was determined as 415 kHz. To our knowledge, this is the first realization of an infrared short‐range communication system based entirely on solution‐processed optoelectronic devices.

## 3 Conclusion

In summary, we have reported a novel technique of blending QDs with controlled mixing of two different matrix QDs to achieve a record high EQE for 1550 nm based CQD LEDs and further improved it by reducing waveguiding optical loss through the utilization of hemispherical lens. The device efficiency competes favorably with commercially available LEDs emitting at 1550 nm (Table S3, Supporting Information) based on III‐V InGaAs. Moreover, we have studied the potential of these devices for the application in short‐range infrared communications. We have reported modulation bandwidth in the megahertz range and demonstrated an all‐CQD‐based integrated system comprising a LED and a photodiode, for short‐range infrared communications.

## 4 Experimental Section

### Synthesis of PbS QDs

1.75, and 1.35 eV excitonic peak based PbS QDs, used as the matrix materials for the devices, were synthesized using standard Schlenk technique. In brief, 0.446 g lead oxide (PbO), 1.6 mL oleic acid, and 18 mL 1‐octadecene (ODE) were pumped for 1 h at 100 °C. For 1.75 eV PbS QDs, the temperature was set to 80 °C and 1 mmol hexamethyldisilathiane (HMS) mixed with 5 mL ODE was injected immediately. The reaction was quenched with cold acetone after 20 s and the QDs were isolated by precipitation. The QDs were further purified by dispersion/precipitation with toluene/acetone 3 times. For 1.35 eV QDs, the temperature was set at 80 °C and right after the injection, the heating was stopped (without removing the heating mantle) and the solution was allowed to cool down gradually (≈1 h).

0.79 eV excitonic peak based PbS QDs were synthesized by a previously reported multi‐injection method with modification.^[^ ^27^ ^]^ Typically, 0.446 g PbO, 3.8 mL oleic acid, and 50 mL ODE were mixed together at 100 °C under vacuum for 1 h. Once under argon, a solution of 65 µL HMS in 3 mL ODE was quickly injected. After 6 min, a second injection of 80 µL of HMS in 9 mL of ODE was dropwise injected and then, the heating was stopped immediately and the solution was cooled down gradually to room temperature. The QDs were purified three times with a mixture of acetone/ethanol, redispersing in toluene. The final concentration of all the PbS QDs solutions was adjusted to 30 mg mL^−1^ before the LED device preparation.

### ZnO NC Synthesis

ZnO NCs were synthesized following standard method as described in the authors' previous report.^[^ ^17^ ^]^ 2.95 g zinc acetate dihydrate was dissolved in 125 mL methanol solvent and the temperature of the reaction bath was set to 60 °C. In a separate vial, 1.48 g potassium hydroxide (KOH) (85%) flakes were dissolved in 65 mL methanol. Under continuous stirring, the KOH solution was then added dropwise to the zinc acetate solution for a time span of 4–5 min while the reaction temperature was fixed at 60 °C. After 2.5 h, the heating source was removed, and the reaction bath was allowed to cool down to room temperature. The solution was then transferred to centrifuge tubes and centrifuged at 5000 rpm for 5 min. The supernatants were then discarded and remnants were washed again with methanol following the similar procedure and finally dried in nitrogen flow to get ZnO NCs. For electron injection layer, ZnO NCs were dispersed in a solution of 2% butylamine in chloroform with a concentration of 80 mg mL^−1^.

### LED Device Preparation

LEDs were prepared on pre‐cleaned ITO coated glass as described in the authors’ previous report.^[^ ^16^ ^]^ ZnO NCs in chloroform (80 mg mL^−1^) was spun on top of the substrate with a spin speed of 4000 rpm to form the electron injection layer of ≈80 nm. The active layer was deposited on top of the ZnO layer. For blended devices, all QDs (1.75, 1.35, and 0.79 eV excitonic peak based PbS) were prepared in separate vials with the same concentration (30 mg mL^−1^) before mixing. For binary blends, emitter PbS QDs were mixed to matrix PbS QDs as 7.5% mixing ratio. For blended matrix devices, 1.35 and 1.75 eV exciton peak based QDs were mixed with different mixing ratios. Then, the blended matrix QDs were added to emitter QDs so that in the final solution 7.5% emitter QDs were present. Zinc iodide (ZnI_2_) and 3‐mercaptopropionic acid (MPA) were mixed to use as ligand for solid state QD film formation as described elsewhere.^[^ ^28^ ^]^ The thickness of the active layer was adjusted as ≈60 nm by repeating the ligand treatment procedure three times. The hole‐transporting layer of ≈50 nm was formed using the PbS QDs (1.35 eV PbS QD) treated with 0.02% 1,2‐ethanedithiol (EDT) in acetonitrile solution. The top electrode was formed by thermally evaporated Au deposition through a pre‐patterned shadow mask (Nano 36 Kurt J. Lesker) at a base pressure of 10^−6^ mbar. The active area of each device was 3.14 mm^2^.

### LED Performance Characterization

All the devices were fabricated and characterized in ambient air conditions. Current density–voltage (*J*–*V*) characteristics were recorded using a computer‐controlled Keithley 2400 source measurement unit. EQE of the devices was calculated by detecting the radiance from the device with a calibrated Newport 918D‐IR‐OD3 germanium photodetector connected to Newport 1918‐C power meter in parallel to the *J*–*V* measurements and further confirmed with a Newport 818 IG InGaAs photodetector. Lambertian emission was assumed during radiance calculation. The thickness of the glass substrate was considered during the solid angle calculation. For half‐ball lens attached devices, the lenses (NBK‐7 from Edmund Optics) were attached to the glass side of the LED devices. Norland UV curing optical adhesive was used to attach the lens with the glass substrate. The emitted power of lens attached LEDs was measured with a calibrated integrating sphere (IS210C from Thorlab).

### Photodiodes Preparation

The photodiodes were prepared on top of ITO coated glass. ZnO layer was formed on top of ITO using already prepared ZnO NCs in chloroform (40 mg mL^−1^) spun with 4000 rpm for 30 s and subsequently baked for 30 min on top of hot plate at 250 °C. The active layer was prepared with 0.79 eV excitonic peak based QDs treated with ZnI_2_/MPA based mixed ligands. The active layer thickness was adjusted ≈300 nm. Finally, a 30 nm hole transporting layer was formed with 1.75 eV excitonic peak based QDs treated with EDT with aforementioned procedure. Thermally evaporated Au with active area of 3.14 mm^2^ was used as the top electrodes.

### Photodiode Characterizations

Current density–voltage (*J*–*V*) characteristics were performed with Keithley 2400 source measurement unit. A supercontinuum laser (NKT Photonics) with varying intensity was used as the light source (*λ* = 1560 nm) for light response measurements. The EQE of the device was measured using an in‐house built integrated system. A chopped monochromatic illumination was used as the light source (chopping frequency 220 Hz). The source wavelength was scanned over 350 to 1700 nm range. The device photocurrent response of the chopped signal was measured using a Stanford Research system lock‐in amplifier (SR830). A Stanford Research system low noise current preamplifier (SR570) was used in between the sample and the lock‐in‐amplifier to amplify the signal.

### Modulation Measurements and Integrated Device Characterizations

To characterize the modulation bandwidth, the LEDs were driven with a DC power source connected with a function generator through bias‐tee. The frequency of the signal was varied according to the experimental needs. The sample response was measured with an InGaAs avalanche photodetector (APD430C/M from Thorlab). The frequency modulation of the integrated devices was measured following the aforementioned method using PbS QD photodiode as signal receiver.

### Photoluminescence and Electroluminescence Measurements

Photoluminescence (PL) and quantum yield (PLQY) measurements were performed utilizing a Horiba Jobin Yvon iHR550 Fluorolog system coupled with a Hamamatsu RS5509‐73 liquid‐nitrogen cooled photomultiplier tube and a calibrated Spectralon‐coated Quanta‐phi integrating sphere. A Vortran Stradus 637 nm continuous wave laser was used as the excitation source for all the steady state measurements. The PL spectra were corrected taking into account the system response function. The detail of the PLQY measurements was described elaborately in the authors’ previous report.^[^ ^16^ ^]^ Electroluminescence spectra were collected using an ANDOR InGaAs array CCD camera. The voltage bias to the device for electroluminescence measurement was applied with a Keithley 2400 source meter. For imaging surface of the devices, a SWIR lens was attached to NIT‐WiDy‐SenS‐320V‐ST InGaAs camera (Iberoptics Sistemas Ópticos), where the pictures and videos were recorded ≈20 cm away from the sample surface.

## Conflict of Interest

The authors declare no conflict of interest.

## Author Contributions

G.K. co‐developed the concept designed the experiments and directed the study. S.P. co‐developed the concept, fabricated, and characterized the devices, and analyzed the data. M.D. synthesized the QDs. B.K. helped in photodiode characterizations. N.T. has performed the optical simulations and captured the photo/video of LED emission with infrared camera. S.P. and G.K. wrote the manuscript with input from co‐authors.

## Supporting information

Supporting Information

## Data Availability

The data that support the findings of this study are available from the corresponding author upon reasonable request.

## References

[1] F. P. García de Arquer , D. V. Talapin , V. I. Klimov , Y. Arakawa , M. Bayer , E. H. Sargent , Science 2021, 373, eaaz8541. 34353926 10.1126/science.aaz8541

[2] J. Huang , J. Lee , J. Vollbrecht , V. V. Brus , A. L. Dixon , D. X. Cao , Z. Zhu , Z. Du , H. Wang , K. Cho , G. C. Bazan , T. Nguyen , Adv. Mater. 2020, 32, 1906027. 10.1002/adma.201906027 31714629

[3] X. Tang , M. M. Ackerman , P. Guyot‐Sionnest , Laser Photonics Rev. 2019, 13, 1900165.

[4] S. Yakunin , B. M. Benin , Y. Shynkarenko , O. Nazarenko , M. I. Bodnarchuk , D. N. Dirin , C. Hofer , S. Cattaneo , M. V. Kovalenko , Nat. Mater. 2019, 18, 846. 31263225 10.1038/s41563-019-0416-2

[5] L. Pérez , Í. Rodríguez , N. Rodríguez , R. Usamentiaga , D. F. García , Sensors 2016, 16, 335. 10.3390/s16030335 PMC4813910 26959030

[6] S. J. Sweeney , A. F. Phillips , A. R. Adams , E. P. O'Reilly , P. J. A. Thijs , IEEE Photonics Technol. Lett. 1998, 10, 1076.

[7] C. Bao , W. Xu , J. Yang , S. Bai , P. Teng , Y. Yang , J. Wang , N. Zhao , W. Zhang , W. Huang , F. Gao , Nat. Electron. 2020, 3, 156. 32226921 10.1038/s41928-020-0382-3 PMC7100905

[8] K. Yoshida , P. P. Manousiadis , R. Bian , Z. Chen , C. Murawski , M. C. Gather , H. Haas , G. A. Turnbull , I. D. W. Samuel , Nat. Commun. 2020, 11, 1171. 32127529 10.1038/s41467-020-14880-2 PMC7054290

[9] A. Ren , H. Wang , W. Zhang , J. Wu , Z. Wang , R. V. Penty , I. H. White , Nat. Electron. 2021, 4, 559.

[10] M. Liu , N. Yazdani , M. Yarema , M. Jansen , V. Wood , E. H. Sargent , Nat. Electron. 2021, 4, 548.

[11] A. P. Litvin , I. V. Martynenko , F. Purcell‐Milton , A. V. Baranov , A. V. Fedorov , Y. K. Gun'ko , J. Mater. Chem. A 2017, 5, 13252. 10.1039/c7tb01425b 32264322

[12] X. Dai , Z. Zhang , Y. Jin , Y. Niu , H. Cao , X. Liang , L. Chen , J. Wang , X. Peng , Nature 2014, 515, 96. 25363773 10.1038/nature13829

[13] Y.‐H. Won , O. Cho , T. Kim , D.‐Y. Chung , T. Kim , H. Chung , H. Jang , J. Lee , D. Kim , E. Jang , Nature 2019, 575, 634. 31776489 10.1038/s41586-019-1771-5

[14] H. Shen , Q. Gao , Y. Zhang , Y. Lin , Q. Lin , Z. Li , L. Chen , Z. Zeng , X. Li , Y. Jia , S. Wang , Z. Du , L. S. Li , Z. Zhang , Nat. Photonics 2019, 13, 192.

[15] Q. Su , H. Zhang , S. Chen , npj Flexible Electron. 2021, 5, 8.

[16] S. Pradhan , F. Di Stasio , Y. Bi , S. Gupta , S. Christodoulou , A. Stavrinadis , G. Konstantatos , Nat. Nanotechnol. 2019, 14, 72. 30510279 10.1038/s41565-018-0312-y

[17] S. Pradhan , M. Dalmases , A. Baspinar , G. Konstantatos , Adv. Funct. Mater. 2020, 30, 2004445.

[18] M. Vasilopoulou , H. P. Kim , B. S. Kim , M. Papadakis , A. E. Ximim Gavim , A. G. Macedo , W. Jose da Silva , F. K. Schneider , M. A. Mat Teridi , A. G. Coutsolelos , A. R. bin Mohd Yusoff , Nat. Photonics 2020, 14, 50.

[19] X. Gong , Z. Yang , G. Walters , R. Comin , Z. Ning , E. Beauregard , V. Adinolfi , O. Voznyy , E. H. Sargent , Nat. Photonics 2016, 10, 253.

[20] L. Gao , L. N. Quan , F. P. García de Arquer , Y. Zhao , R. Munir , A. Proppe , R. Quintero‐Bermudez , C. Zou , Z. Yang , M. I. Saidaminov , O. Voznyy , S. Kinge , Z. Lu , S. O. Kelley , A. Amassian , J. Tang , E. H. Sargent , Nat. Photonics 2020, 14, 227.

[21] S. Pradhan , M. Dalmases , G. Konstantatos , Adv. Mater. 2020, 32, 2003830. 10.1002/adma.202003830 32996211

[22] K. Tuong Ly , R.‐W. Chen‐Cheng , H.‐W. Lin , Y.‐J. Shiau , S.‐H. Liu , P.‐T. Chou , C.‐S. Tsao , Y.‐C. Huang , Y. Chi , Nat. Photonics 2017, 11, 63.

[23] R. Yu , F. Yin , X. Huang , W. Ji , J. Mater. Chem. C 2017, 5, 6682.

[24] Q. Zhang , X. Gu , Z. Chen , J. Jiang , Z. Zhang , J. Wei , F. Li , X. Jin , Y. Song , Q. Li , Opt. Express 2017, 25, 17683. 28789260 10.1364/OE.25.017683

[25] H.‐Y. Lan , I.‐C. Tseng , H.‐Y. Kao , Y.‐H. Lin , G.‐R. Lin , C.‐H. Wu , IEEE J. Quantum Electron. 2018, 54, 1.

[26] M. Vafaie , J. Z. Fan , A. M. Najarian , O. Ouellette , L. K. Sagar , K. Bertens , B. Sun , F. P. G. de Arquer , E. H. Sargent , Matter 2021, 4, 1042.

[27] J. W. Lee , D. Y. Kim , S. Baek , H. Yu , F. So , Small 2016, 12, 1328. 26763178 10.1002/smll.201503244

[28] S. Pradhan , A. Stavrinadis , S. Gupta , Y. Bi , F. Di Stasio , G. Konstantatos , Small 2017, 13, 1700598. 10.1002/smll.201700598 28401651

