# Supplementary material for: Colloidal Quantum Dot Light Emitting Diodes at Telecom Wavelength with 18% Quantum Efficiency and Over 1 MHz Bandwidth
Source: Adv Sci (Weinh). 2022 May 4;9(20):2200637. doi: 10.1002/advs.202200637 (PMC9284174; doi:10.1002/advs.202200637)
Supplement: Supplementary file 1 — Supporting Information [file ADVS-9-2200637-s001.pdf]

ADVANCED SCIENCE
Open Access

Supporting Information

for *Adv. Sci.*, DOI 10.1002/advs.202200637

Colloidal Quantum Dot Light Emitting Diodes at Telecom Wavelength with 18% Quantum Efficiency and Over 1 MHz Bandwidth

*Santanu Pradhan, Mariona Dalmases, Nima Taghipour, Biswajit Kundu and Gerasimos Konstantatos**

Supporting information for

Colloidal Quantum Dot Light Emitting Diodes at Telecom Wavelength with 18% Quantum Efficiency and over 1 MHz bandwidth

Santanu Pradhan[1], Mariona Dalmases[1], Nima Taghipour[1], Biswajit Kundu[1] and Gerasimos Konstantatos[1, 2*]

[1]ICFO-Institut de Ciències Fotòniques, The Barcelona Institute of Science and Technology, 08860 Castelldefels (Barcelona), Spain

[2]ICREA—Institució Catalana de Recerca i Estudis Avançats, Passeig Lluís Companys 23, 08010 Barcelona, Spain

* gerasimos.konstantatos@icfo.es

S1: Device performance statistics:

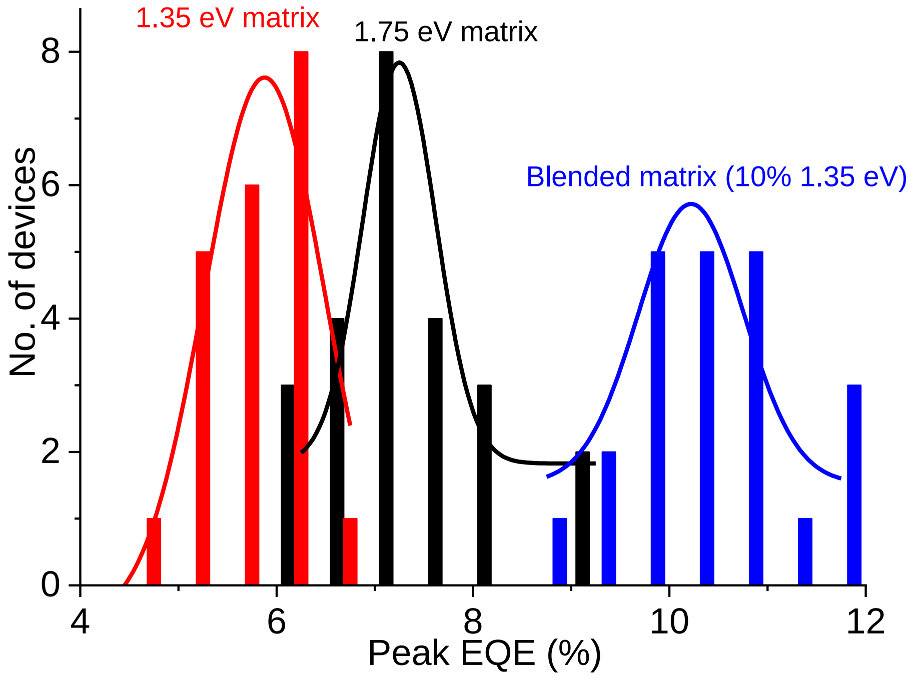

Figure S1: The device performance statistics for 1.35 eV matrix, 1.75 eV matrix based binary and 10% 1.35 eV QDs + 90% 1.75 eV QDs blended matrix-based devices. 21-22 devices were measured for each case.

S2: Effect of mix matrix ratio on the device performance:

a.

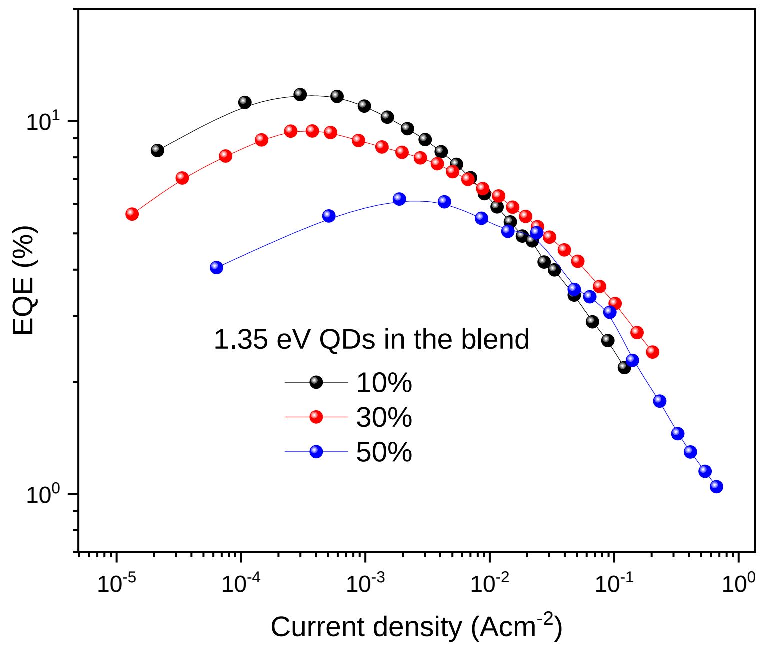

b.

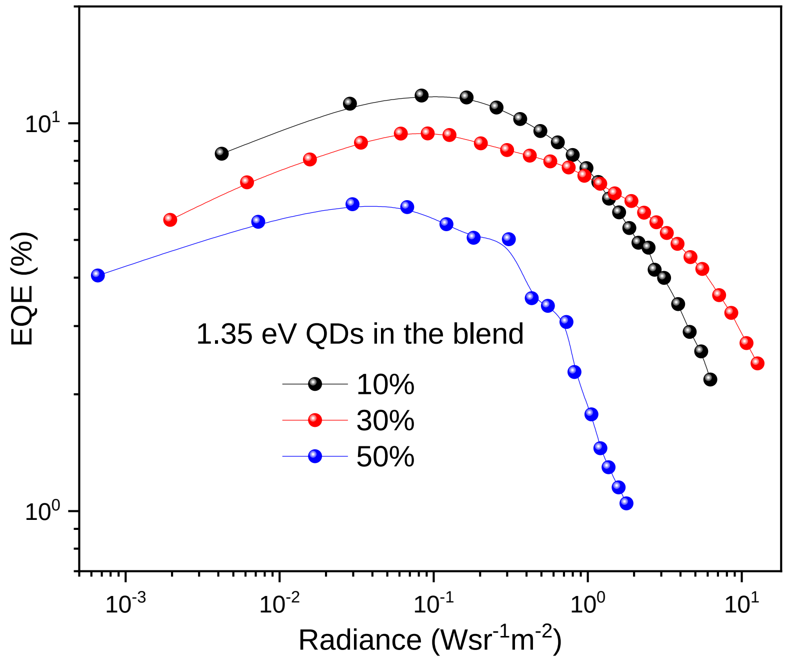

Figure S2: (a) EQE as a function of injected current density with the variation of blend ratio of the QDs in the matrix. (b) EQE as a function of radiance. The performance goes down with higher loading of 1.35 eV QDs in the blend.

S3: Effect of low loading of 1.35 eV QDs in mixing on device performance:

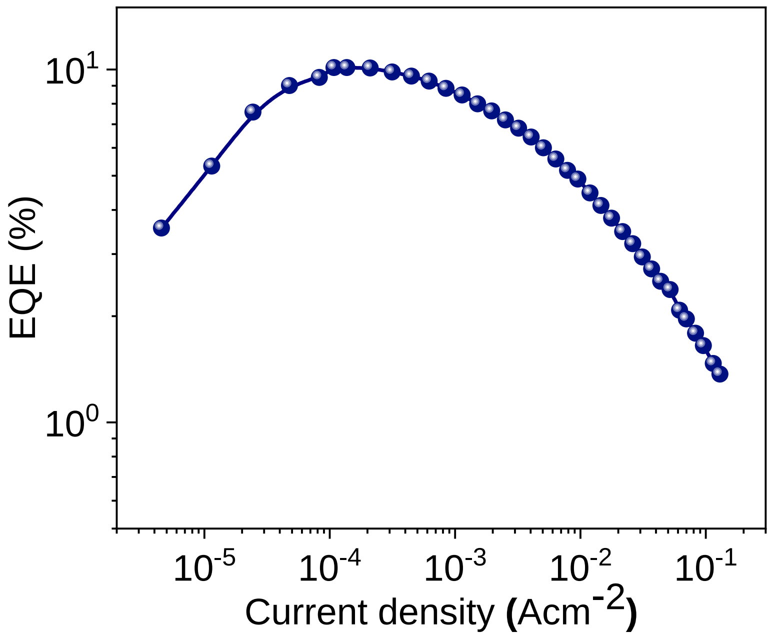

Figure S3: EQE spectra of 5% 1.35 eV QD loading for mixed matrix-based device

S4: Lumerical FDTD simulation to estimate the waveguide loss in the glass substrate:

For full electromagnetics simulation, we used Commercial software, Ansys Lumerical Finite Difference Time Domain (FDTD). The full stack LED device was placed a top of a glass substrate. To calculate the transmitted light in the glass side (bottom of the LED structure), a dipole box with random dipole orientation was employed to model the active medium. All layers of LED are assumed to be homogenous medium for which the refractive indices were extracted from ellipsometry measurement. Perfectly matched layers boundary conditions are used at $\pm y$ and periodic boundary conditions are used at $\pm x$ direction. The field are recorded using frequency-domain filed and located at the bottom of the structure after glass substrate normal to the y-direction. The fraction of transmitted power is calculated based on the transmitted power in monitor divided by total emitted light from the dipole box.

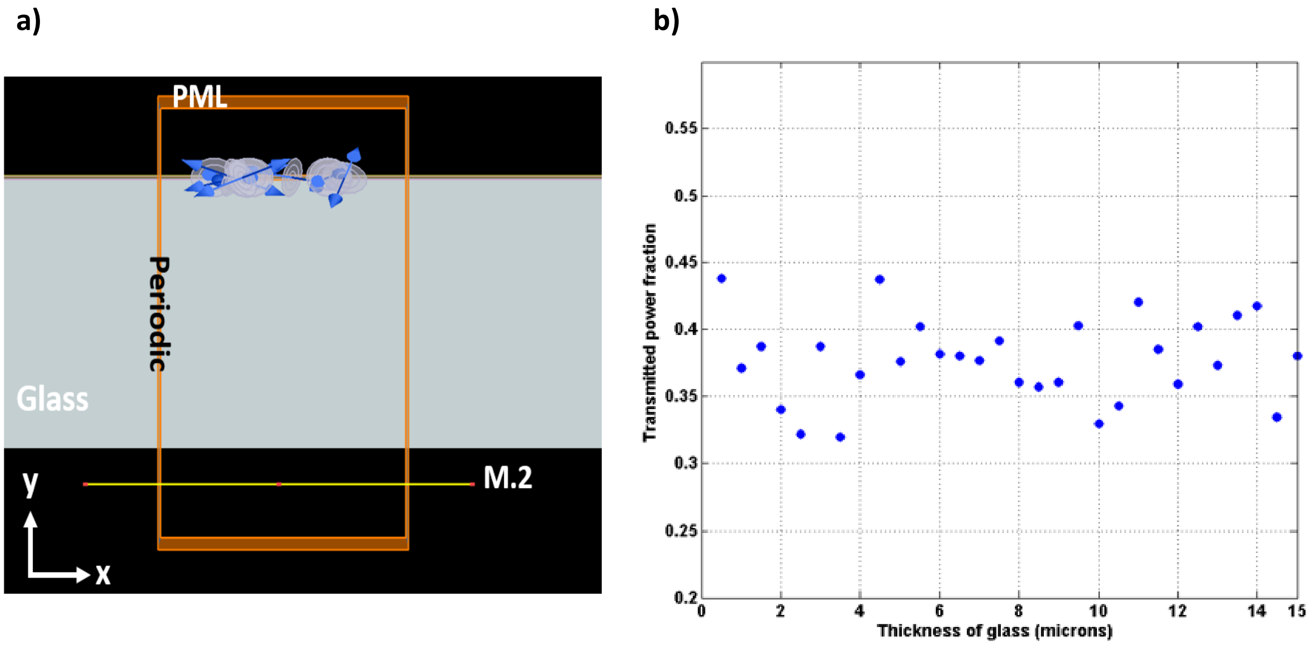

Figure S4: (a) Schematic of the stack used for numerical simulation. (b) The simulation shows on an average 40% transmission of light from the glass substrate indicating nearly 60% of light loss from glass and other layers.

S5: Hemispherical lens attachment to the LED substrate to reduce optical waveguide loss:

The finite thickness of the ITO coated glass substrate, the difference in refractive index between glass and LED materials contribute to optical waveguide loss through all sides of the device. Organic and colloidal quantum dot based visible LEDs based on similar device structure showed remarkable improvement while using hemispherical lens to reduce the optical loss [1, 2]. The schematic of the lens attached glass substrate is shown in Fig. S5 (a).

a.

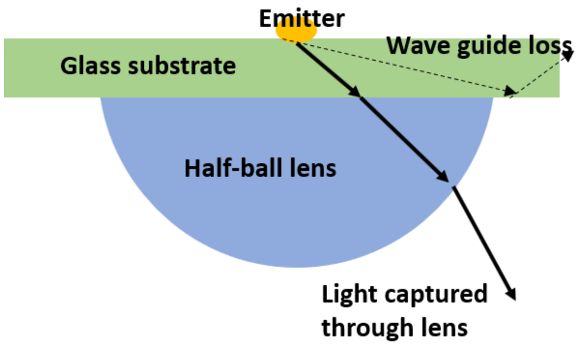

b.

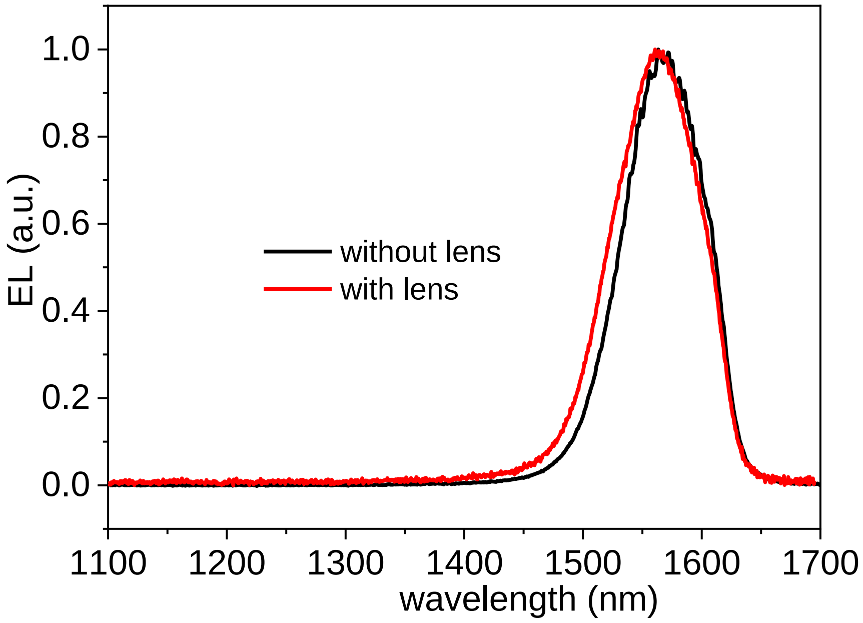

Figure S5: (a) The schematic of the hemispherical lens attached device. The dashed line shows the path of waveguide loss. The solid path shows how incorporation of hemispherical lens reduces the optical loss. (b) Electroluminescence spectra of LED devices with and without lens. The use of half-ball lens does not change the spectral shape of emission.

S6. Photoluminescence properties of the active materials:

a)

b)

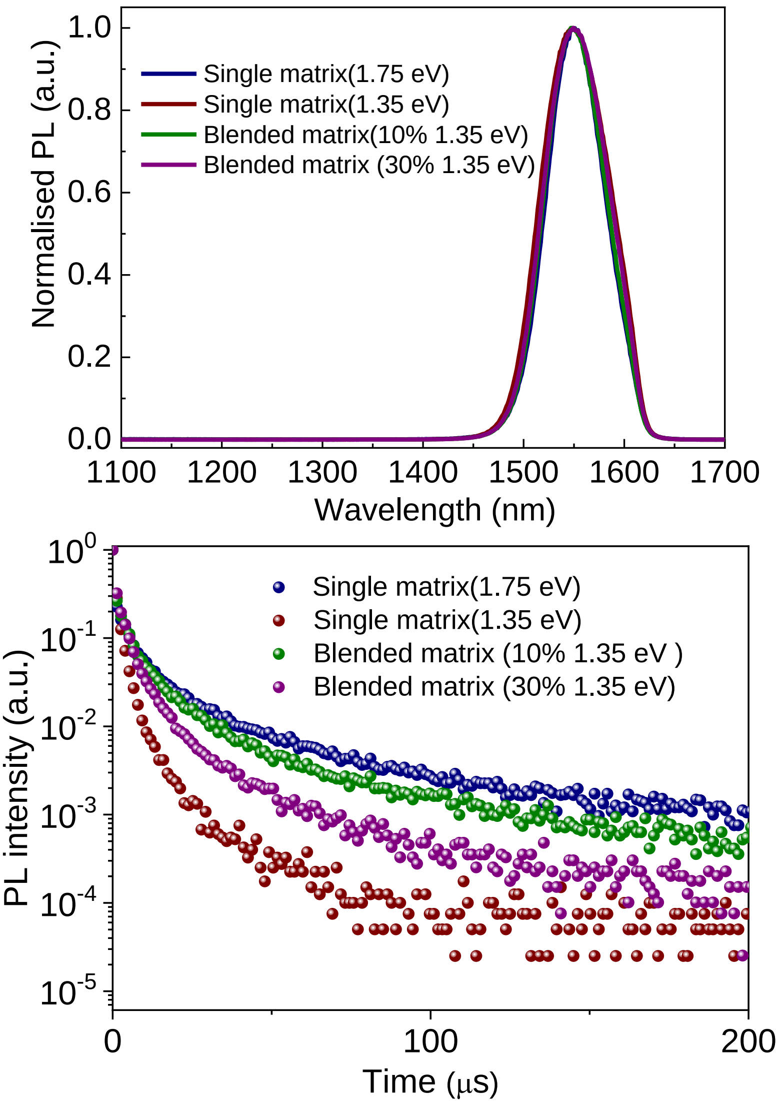

Figure S6: (a) Photoluminescence spectra of the different matrix compositions. For all the cases, the emission is observed around 1550 nm corresponding to the bandgap of the emitter QDs. (b) The slower component of the photoluminescence decay curve varies as a function of the matrix compositions as summarized in table S1. The photoluminescence decay time can be tuned with the blending ratio in the blended matrix-based devices.

**Sample (matrix QD)**	**τ_1 (μs)**	**τ_2 (μs)**
1.75 eV	0.59	8.56

1.35 eV	0.72	2.99
10% matrix mix	0.63	6.84
30% matrix mix	0.65	4.56

Table S1: Photoluminescence time decay parameters of different matrix based blended solids.

S7: The schematic of the heterojunctions formed in different single matrix or blend matrix based QD ensembles:

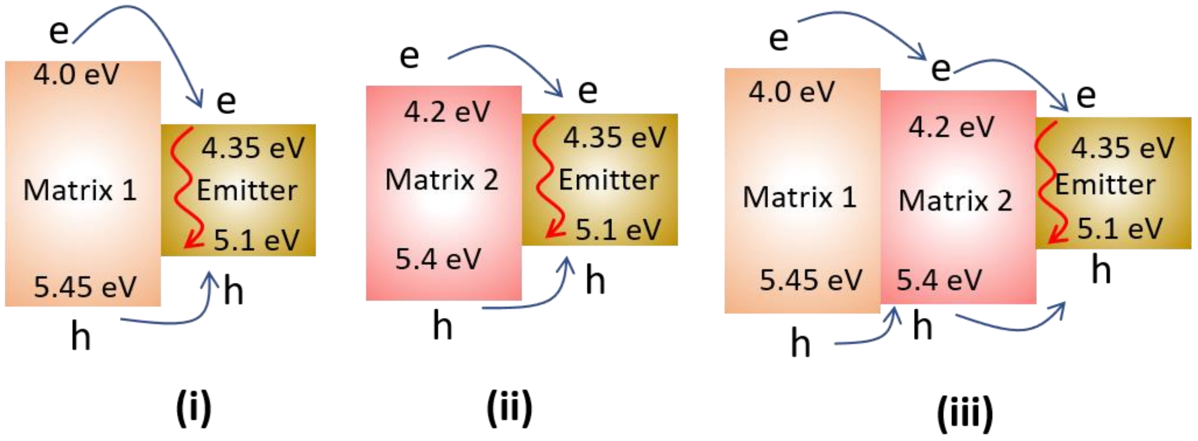

Figure S7: The schematic of the heterojunction formed in the active material of (i) 1.75 eV QD single matrix, (ii) 1.35 eV QD single matrix, (iii) 1.75 eV, 1.35 eV QDs blended matrix-based devices. The energy values are taken from our previous reports [3-5].

S8. Three-dimensional packing of colloidal quantum dot in binary and mix-matrix mixing and prediction of dot-to-dot hopping probability:

The average diameter of the emitter QDs is 6 nm (estimated from excitonic absorption peak at 0.79 eV).

Considering the QDs as perfect sphere Emitter QD Volume will be: $V \approx \frac{4\pi}{3}\left(3 \times 10^{-7}\right)^{3} \text{cm}^{3}$

So, the number of No. of emitter QD per 1 cm^3 volume (N) $\sim \frac{0.74}{V} \sim 6.5 \times 10^{18}$ cm^{-3}.

Considering the QDs are distributed in isotropic way upon mixing as shown in Fig. S5, we can estimate the volume which contain each of the QD in the matrix.

In 7.5% binary mixing, the number of emitters QDs per 1 cm^3 will be 4.9×10^{17} cm^{-3}

The volume of the cube containing each of the emitter QD as shown in Fig. S8 can be calculated as, $1/(4.9 \times 10^{17}) \approx 2 \times 10^{-18} \text{cm}^{3}$
The edge of each cube will be $\sim \sqrt[3]{2 \times 10^{-18}} \sim 12.8\ nm$

So, the centre-to-centre distance between two emitter QD in the matrix is $d_{th} \sim 12.8\ nm$.

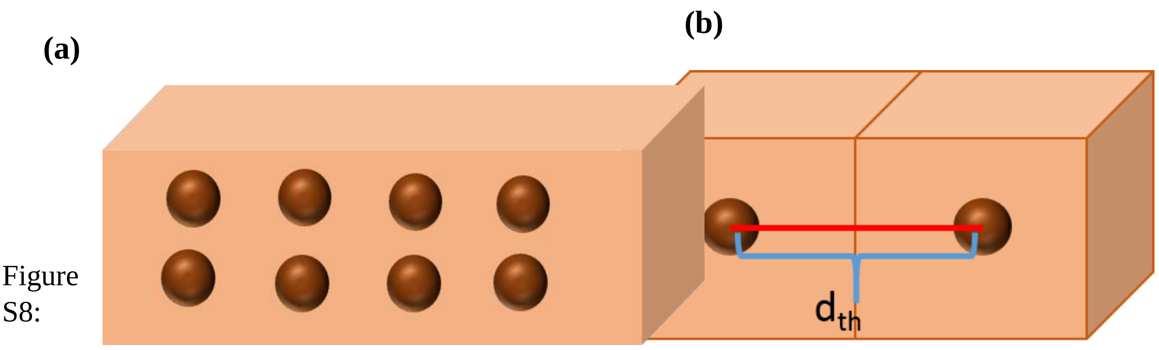

Figure S8: (a) Schematic of isotropic Emitter QD distribution in matrix; (b) Centre-to-centre distance between two nearest Emitter QDs.

Considering a similar approach for the 900 nm (1.37 eV) excitonic peak based QDs, we can estimate the dot-to-dot distance between the 900 nm dots is 5.8 nm. Thus, the average distance an electron should hop from the 900 nm dot to another 900 nm dot or 1550 nm emitter dot should be around 5.8 nm. Utilizing these data one can estimate the hopping probability of electron transmission.

Hopping Probability:

The hopping probability can be given by the Miller-Abrahams expression [6],

$$P \propto \exp\left(\frac{-2R}{a} - \frac{\Delta E}{kT}\right)$$

Here, *R* is the hopping distance, *a* is the carrier localization, *ΔE* is the activation energy for the hopping process. *kT* is the thermal energy.

Hopping probability of electrons from 900 nm QD to 900 nm/1550 nm QD:

The average dot-to-dot distance should be taken as the hopping distance which is given by 5.8 nm. The localization length should be the diameter of 900 nm QD (~2.9 nm). The activation energy for this case is 0 eV. Thus, the Hopping probability can be estimated as,

$$P_1 \propto \exp(\frac{-11.6}{2.9})$$

Hopping probability of electrons from 900 nm QD to 700 nm QD:

The average dot-to-dot distance should be taken as the hopping distance as given by 2.1 nm. The localization length should be the diameter of 900 nm QD (~2.9 nm). The activation energy for this case should be the conduction band offset between 900 nm and 700 nm QDs (0.15 eV). Thus, the Hopping probability can be estimated as,

$$P_2 \propto \exp(\frac{-4.2}{2.9} - \frac{0.15}{0.026})$$

One can estimate (P_1/P_2) as *exp(3.5)*. P_1 is around 31 times more probable than P_2. Thus, in the mix matrix blend, hopping probability from 900 to 900 nm QDs or the emitter QDs are much higher compared to 900 to 700 nm QDs.

S9: Estimating injection efficiency of mixed matrix devices from SCAPS simulation:

The injection efficiency in an LED is defined by the fraction of charges injected participate in the recombination processes. Thus, the injection efficiency (η_{inj}) can be expressed as,

$$\eta_{inj} = \frac{R_{tot}}{(I/q)}$$

Where R_{tot} is the total recombination rate given by, $R_{tot} = R_{rad} + R_{SRH} + R_{Auger}$. R_{rad} is the radiative recombination rate, R_{SRH} is the trap assisted recombination rate and R_{Auger} is the Auger recombination rate [7]. I is the injection current. The injection efficiency was computed by calculating R_{tot} and I and taking their ratio. Figure S9 shows the variation of R_{tot} and injection current as a function of 1.35 eV QD loading in the blended matrix. The injection efficiency shows improvement with low loading (1-10%) and then it starts decreasing with higher 1.35 QD loading in the matrix due to the variation of these parameters. The lowest value is obtained with 70% loading and it starts to grow again up to 100% loading. This variation matches with the obtained device efficiency with 10% loading shows best performance and it goes down with higher loading and 50% loading-based devices showed lower EQE. The injection efficiency linearly proportional with the EQE of the device.

Parameters	ZnO	Emitter PbS QD	Matrix PbS QD (higher bandgap)	Matrix PbS QD (higher bandgap)	EDT treated PbS blocking layer

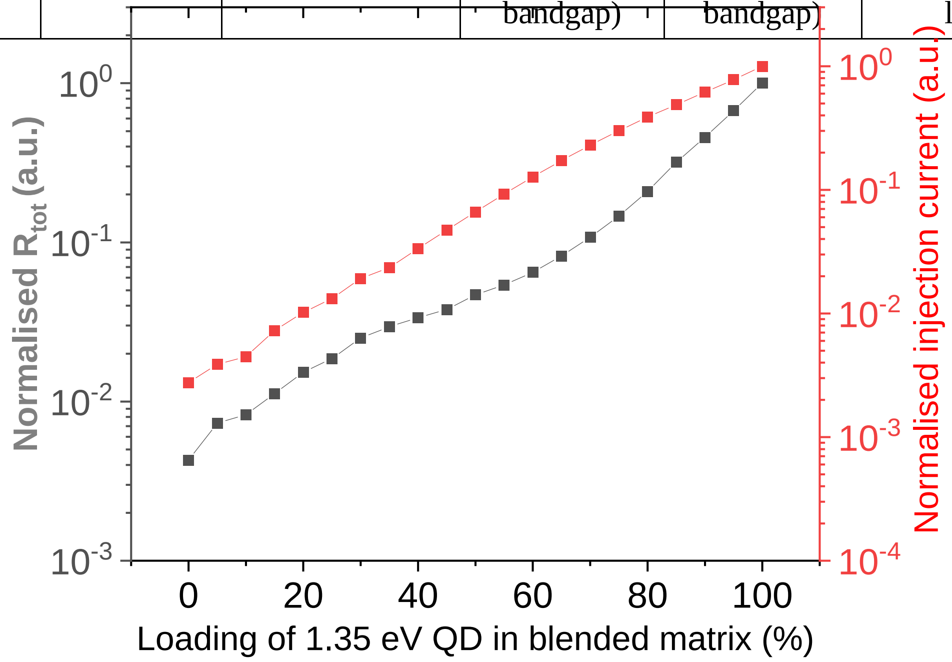

Figure S9: Variation of total recombination rate and injection current as a function of 1.35 eV QD loading in the matrix.

SCAPS simulation:

The binary device structures are considered similar to our previous reports [5, 8]. Figure S9 shows the structure used for SCAPS simulations. For blended matrix, we have taken uniform mixing throughout the matrix. The effective medium approximations for homogeneous mixture were considered. In case of blended matrix, for material properties, uniform (0<y<1) option was chosen. The value of y varied from 0.5 to 0.95 to get the results. The parameters used in the simulation are summarised in Table S2.

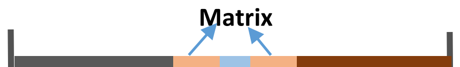

Bandgap (eV)	3.4	0.79	1.45	1.2	1.2
Electron affinity (eV)	4.2	4.35	4.0	4.2	3.8
Relative di electric constant	66	22	22	22	18
CB effective density of states (cm^{-3})	1E+19	1E+19	1E+19	1E+19	9E+18
VB effective density of states (cm^{-3})	1E+19	1E+19	1E+19	1E+19	9E+18
Electron thermal velocity (cm s^{-1})	1E+7	7E+3	7E+3	7E+3	7E+3
Hole thermal velocity (cm s^{-1})	1E+7	7E+3	7E+3	7E+3	7E+3
Electron mobility ($cm^2V^{-1}s^{-1}$)	5E-3	1E-2	1E-2	1E-2	1E-2
Hole mobility ($cm^2V^{-1}s^{-1}$)	5E-3	1E-2	1E-2	1E-2	1E-2
Donor density (cm^{-3})	1E+18	1E+16	1+E16	1+E16	0
Acceptor density (cm^{-3})	0	0	0	0	1E+16
Trap density (cm^{-3})	-	5E+16	5E+16	5E+16	5E+16
Trap depth relative to E_C (eV)	-	0.3	0.3	0.3	0.3
Trap capture cross-section (cm^2)	-	1.2E-13	1.2E-13	1.2E-13	1.2E-13

Table S2: the parameters used for SCAPS simulations

S10. The electroluminescence signal response as a function of applied bias:

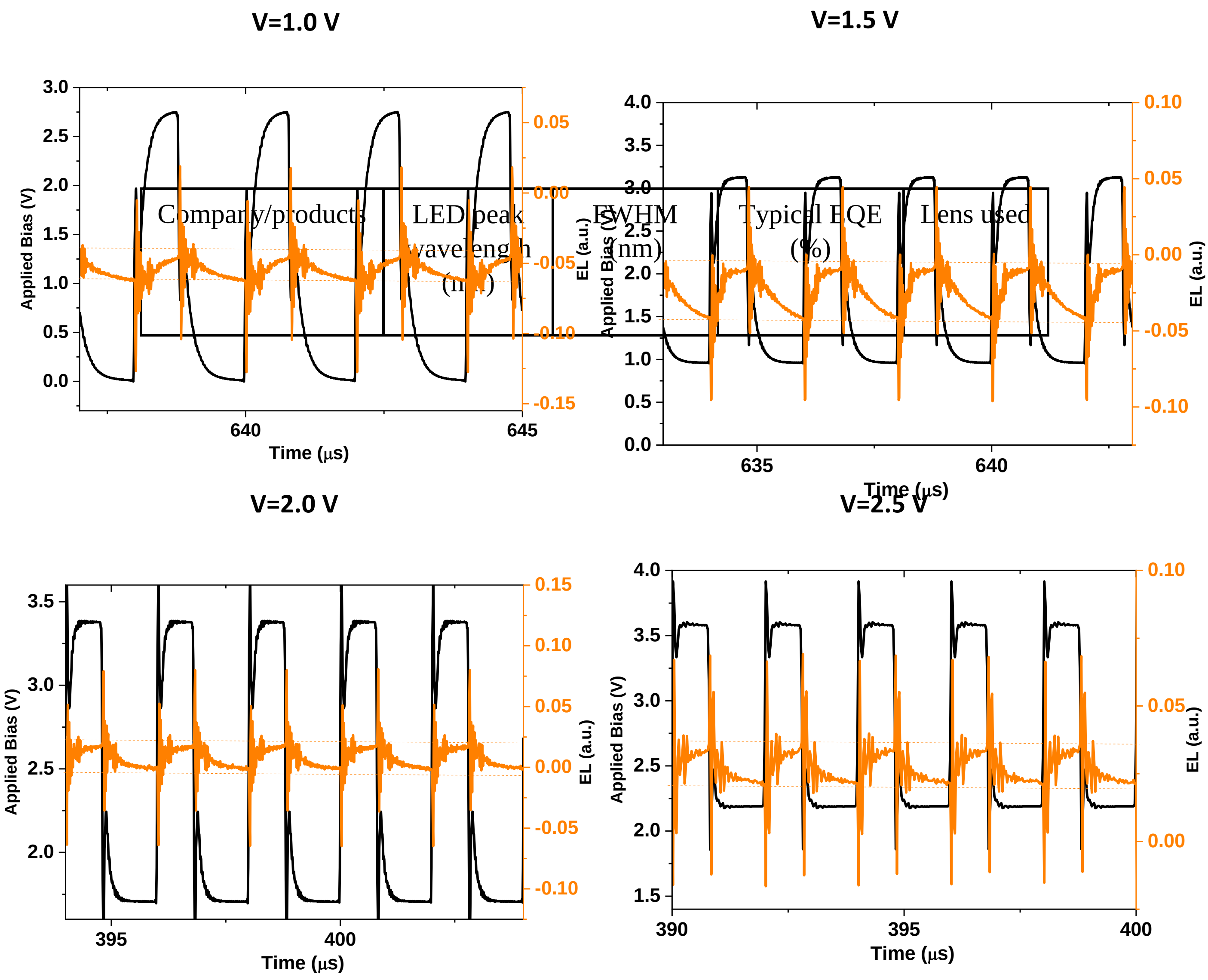

Figure S11: The electroluminescence signal response as captured in the oscilloscope as a function of applied voltage. The applied frequency for the experiment was fixed at f=500 kHz.

S11: Comparison of LED device performance with different commercially available LEDs emitting around 1550 nm:

Company/products	LED peak wavelength (nm)	FWHM (nm)	Typical EQE (%)	Lens used

Thorlab	1550	120	10-11	yes
Ushio	1550	120	9-10	yes
ALPHA-ONE	1550	104	10-11	yes
Hamamatsu	1550	120	8-9.5	yes
Our device	1550	82-86	~11.8 (peak)	No
	1550	82-86	~18.6 (peak)	yes

Table S3: Comparison of 1550 nm emission LED performances.

References:

[1] K. Tuong Ly, R.-W. Chen-Cheng, H.-W. Lin, Y.-J. Shiau, S.-H. Liu, P.-T. Chou, C.-S. Tsao, Y.-C. Huang, Y. Chi, *Nature Photon* **2017**, *11*, 63.
[2] R. Yu, F. Yin, X. Huang, W. Ji, *J. Mater. Chem. C* **2017**, *5*, 6682.
[3] Y. Bi, S. Pradhan, S. Gupta, M. Z. Akgul, A. Stavrinadis, G. Konstantatos, *Adv. Mater.* **2018**, *30*, 1704928.
[4] Y. Bi, A. Bertran, S. Gupta, I. Ramiro, S. Pradhan, S. Christodoulou, S.-N. Majji, M. Z. Akgul, G. Konstantatos, *Nanoscale* **2019**, *11*, 838.
[5] S. Pradhan, M. Dalmases, A. Baspinar, G. Konstantatos, *Adv. Funct. Mater.* **2020**, *30*, 2004445.
[6] N. Vukmirović, L.-W. Wang, *Appl. Phys. Lett.* **2010**, *97*, 043305.
[7] N. Li, K. Han, W. Spratt, S. Bedell, J. Ott, M. Hopstaken, F. Libsch, Q. Li, D. Sadana, *Nat. Photonics* **2019**, *13*, 588.
[8] S. Pradhan, F. Di Stasio, Y. Bi, S. Gupta, S. Christodoulou, A. Stavrinadis, G. Konstantatos, *Nature Nanotech* **2019**, *14*, 72.
